# Oncologic safety of breast conservation following NACT in women with locally advanced breast cancer

**DOI:** 10.3332/ecancer.2023.1554

**Published:** 2023-05-25

**Authors:** Sanjit Kumar Agrawal, Dimple Patel, Pradyumn Shenoy, Rosina Ahmed, Indu Arun, Sanjoy Chatterjee

**Affiliations:** 1 Department of Breast Oncosurgery, Tata Medical Center, Kolkata 700156, India; 2 Department on Oncopathology, Tata Medical Center, Kolkata 700156, India; 3 Department of Radiation Oncology, Tata Medical Center, Kolkata 700156, India

**Keywords:** breast conservation surgery, locally advanced breast cancer, post chemotherapy

## Abstract

**Introduction:**

Breast conservation surgery (BCS) is the accepted standard of treatment for early breast cancer, with evidence from randomized controlled and population-based studies. The oncological outcome of BCS in locally advanced breast cancer (LABC) is mainly available from retrospective series with a small sample size and a shorter follow-up duration.

**Methods:**

A retrospective observational study of 411 non-metastatic LABC patients who received neoadjuvant chemotherapy (NACT) followed by surgery from 2011 to 2016. We retrieved the data from a prospectively maintained database and electronic medical records. Survival data were analyzed by Kaplan–Meier curves and Cox regression using Statistical Package for the Social Sciences 25 and STATA 14.

**Results:**

146/411 (35.5%) women had BCS with a margin positivity rate of 3.42%. With a median follow-up of 64 months (IQR 61, 66), the local relapse rate was 8.9% in BCS and 8.3% after mastectomy. The estimated 5-year locoregional recurrence-free survival (LRFS), recurrence-free survival (RFS), distant disease-free survival (DDFS) and overall survival (OS) rates of BCS were 86.9%, 63.9%, 71% and 79.3%, and 90.1%, 57.9%, 58.3% and 71.5% in the mastectomy group. On univariate analysis, BCS showed superior survival outcomes compared to mastectomy (unadjusted HR (95% CI) for RFS: 0.70 (0.50–1), DDFS: 0.57 (0.39–0.84), OS: 0.58 (0.36–0.93)). After adjusting for age, cT stage, cN stage, poorer chemotherapy response (ypT0/is, N0) and radiotherapy, BCS and mastectomy groups were found comparable in terms of LRFS (HR: 1.1, 0.53–2.3), DDFS (HR: 0.67, 0.45–1.01), RFS (HR: 0.80, 0.55–1.17) and OS (HR: 0.69, 0.41–1.14).

**Conclusion:**

BCS is technically feasible in LABC patients. LABC patients who respond well to NACT can be offered BCS without compromising survival outcomes.

## Introduction

Neoadjuvant chemotherapy (NACT) is the standard treatment for non-metastatic locally advanced breast cancer (LABC). It offers the advantage of downstaging the inoperable disease and increases the prospect of breast conservation surgery (BCS) in patients who would otherwise have been candidates for mastectomy [1–3]. NACT allows the assessment of *in vivo* chemosensitivity, which could guide subsequent drug selection [4]. Furthermore, NACT has also exhibited benefits in de-escalating axillary surgery, with sentinel lymph node biopsy (SLNB) now being the standard for clinically node-negative patients at presentation. The role of SLNB in initially node-positive patients is also being evaluated in the post-NACT setting [5]. Patients treated with NACT have shown to have similar recurrence-free survival (RFS) and overall survival (OS) in comparison with upfront surgery followed by adjuvant therapy, with the added advantage of breast conservation and axilla preservation as described above [6–8].

The safety of BCS in early breast cancer (EBC) has been well established [9]. Despite a mounting rise in the literature that supports the feasibility of BCS in LABC, concerns exist regarding the increased likelihood of locoregional recurrence (LRR) or ipsilateral breast tumour recurrence reported in patients who underwent BCS after NACT in LABC [10–13]. Surgical margins free of tumour have a significant impact on the outcome of BCS, and in some patients, it may be challenging to achieve a clear margin in LABC post-NACT due to a honeycomb pattern response [13].

Unlike EBC, no randomized trial has been conducted to establish the efficacy of BCS in LABC. The only data available is from small observational studies and case series. In India, approximately 29%–52% of women present at stage III [14], with peak age between 40 and 50 years [15]. To date, only one large single-centre study has been published from India addressing the safety of BCS in LABC, which is the common presentation of most patients in lower-middle-income countries. The objective of our study was to compare the survival outcomes of BCS versus mastectomy in LABC post-NACT.

## Patients and methods

**Figure 1.**
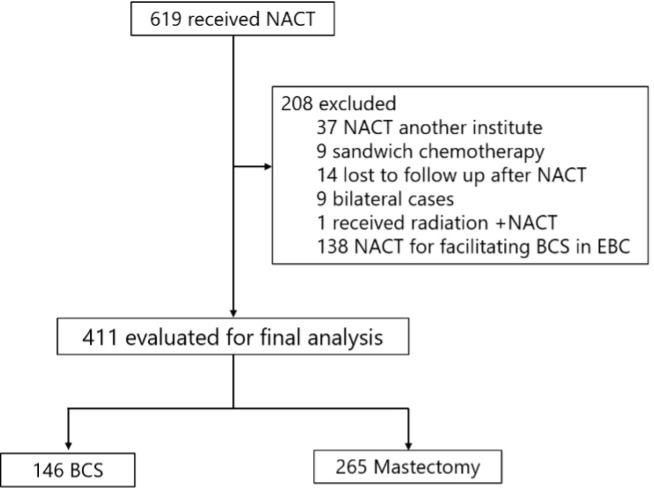
Patient selection. NACT: Neo-adjuvant chemotherapy; BCS: Breast conservation surgery; EBC: Early breast cancer.

A retrospective review of medical records identified 619 patients who received NACT at Tata Medical Center, Kolkata, between January 2011 and December 2016 (ethics committee waiver No: EC/WV/TMC/003/19). 619 patients were analyzed, as illustrated in Figure 1. All the patients underwent core biopsy for diagnosis.

The patients were staged as per clinical and radiological findings before systemic chemotherapy. The fine needle aspiration cytology of the axillary nodes was done only in the clinical normal axilla with suspicious metastatic axillary nodes in the radiological examination. All patients underwent a baseline mammogram, and those planned for BCS also underwent a post-NACT mammogram to assess the response. In addition, before initiating chemotherapy, a metastatic workup (CT thorax and whole abdomen + whole body bone scan) was done in all the patients. The inflammatory breast cancer patients were offered mastectomy only as per the institutional protocol.

The chemotherapy regimen was given as six or eight cycles at intervals of 3 weeks, with the majority receiving anthracycline followed by a taxane. The final decision for the type of surgery was based on the clinical and radiological response. Along with breast surgery, all patients also underwent level 3 axillary dissection. Patients who underwent BCS and had a positive margin on the final histopathology report were treated with cavity excision or completion mastectomy. Per institutional protocol, the surgery was followed by radiation (chest wall/breast and SCF) and adjuvant endocrine/target therapy as per the immunohistochemistry status.

Patients were followed up clinically every 3 months for the initial 2 years and 6 months thereafter, up to 5 years. Subsequently, the follow-up was yearly. Annual mammography was done in all patients.

RFS was defined as the interval between the date of diagnosis and the date of first recurrence or death. Locoregional recurrence-free survival (LRFS) was defined as the interval between the date of diagnosis and the date of local (breast/chest wall/loco-regional) recurrence or death. Distant-disease-free survival (DDFS) was calculated from the date of diagnosis to distant relapse or death. The OS was defined as the interval between the date of diagnosis and the date of death or last known follow-up time.

The data normalcy was checked by the Shapiro–Wilks test. The median (IQR) and percentage were used for summary statistics. We used the Mann–Whitney test to compare continuous variables and the chi-square/Fisher exact test as applicable for categorical variables. Survival data were analyzed by Kaplan–Meier curves and Cox regression. *p* < 0.05 was considered statistically significant. The analysis was done using Statistical Package for the Social Sciences 25 and STATA 14 software.

## Results

411 patients with LABC who received NACT were included in the analysis. The median age was 49.2 years (IQR 43, 56), and BMI was 26 kg/m^2^ (IQR 23.1, 29.7). Invasive ductal carcinoma comprised 96.3% of cases. At presentation, 261 (64.1%) had T4 disease, whereas 124 (30.1%) had T3 disease. 269 (65.4%) patients were N1, and 105 (25.6%) were N2–N3. 360 (87.6%) patients received anthracycline and taxane, while 31 (7.5%) received anthracycline only. 146 (35.5%) patients were treated with BCS with axillary dissection, and 265 (64.6%) underwent modified radical mastectomy (MRM). For BCS, the margin positivity rate was 3.42% (Table 1).

Comparing the two groups (Table 2), the BCS patients were younger than those who had a mastectomy (45.1 versus 50 years). Mastectomy patients had higher T stage (T4: 70.6 versus 52.1%) and N stage and had a poorer chemotherapy response (pCR 14.3 versus 22.6%) compared to those who had BCS, as summarized in Table 2.

### Follow-up and survival analysis

**Figure 2.**
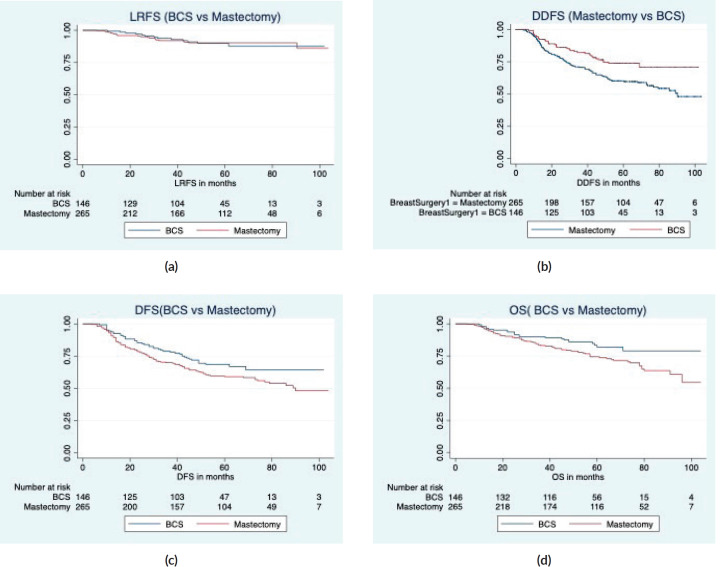
Survival comparison (Kaplan–Meier graph) between BCS versus mastectomy in LABC patients. (a): LRFS comparison between BCS and mastectomy. (b): DDFS comparison between BCS and mastectomy. (c): RFS comparison between BCS and mastectomy. (d): OS comparison between BCS and mastectomy.

With a median follow-up of 64 months (IQR 61, 66), there were 7 local, 6 regional and 36 distant relapses in the BCS group, and 5 local, 17 regional and 106 distant relapses in the mastectomy group (Table 3). The estimated 5-year LRFS, RFS, DDFS and OS rates of the BCS group were 86.9%, 63.9%, 71% and 79.3%, respectively, and those of the mastectomy group were 90.1%, 57.9%, 58.3 and 71.5%, respectively (Figure 2). On univariate analysis, BCS showed superior survival outcomes compared to mastectomy (unadjusted HR (95% CI) for RFS: 0.70 (0.50–1), DDFS: 0.57 (0.39–0.84) and OS: 0.58 (0.36–0.93)). After adjusting for age, cT stage, cN stage, PCR (ypT0/is, N0) and radiotherapy, BCS and mastectomy groups were found equivalent in terms of LRFS (HR: 1.1, 0.53–2.3), DDFS (HR: 0.67, 0.45–1.01), RFS (HR:0.80, 0.55–1.17) and OS (HR:0.69, 0.41–1.14) (Table 4).

## Discussion

NACT has a proven advantage in enabling BCS in patients who were not suitable candidates during the initial presentation. Our study evaluated the local recurrence rates of BCS compared with mastectomy in patients with LABC who received NACT. The BCS in selected LABC patients has shown equivalent survival outcomes in our cohort.

The breast cancer patients subjected to NACT may show a honeycomb pattern response preventing an accurate assessment of surgical margins. Thus, concern regarding the increased likelihood of recurrence in LABC patients treated with BCS after NACT is a major deterrent in the application of BCS in advanced breast cancer. Some studies have reported higher recurrence rates; however, for a large percentage of these patients, radiation therapy was the only local-regional treatment without an attempt to resect the primary tumour site, and some included patients with inflammatory carcinoma [16–18]. In contrast, other recent studies have not found any significant difference in local recurrence rate following BCS in LABC patients [19–22].

In our study, the LR was 8.9% for BCS and 8.3% for mastectomy. The results are comparable to the study by Chou *et al* [23] (*N* = 1,047, 59.2 months follow-up), with an LRR of 8.8% in the BCS group and 10.7% in the mastectomy group. Similarly, a meta-analysis of eight trials published in 2016 also showed a local recurrence rate of 9.2% in the BCS versus 8.3% in the mastectomy (odds ratio 1.07, 95% confidence interval 0.28–1.48; *p* = 0.66) [20].

The EBC Trialists’ Collaborative Group meta-analysis reported that NACT was associated with more frequent local recurrence in BCS patients (15-year LRR 21.4% for NACT versus 5.9% for adjuvant chemotherapy). The authors concluded that the likely reason for this was an increase in BCS rate in women who responded well to NACT. However, patients included in the meta-analysis were treated between 1983 and 2002 and have received chemotherapy regimens that have now been abandoned. Only 18·9% had received taxane-based chemotherapy, while anti-HER2 drugs were unavailable. For some patients with complete clinical responses, surgery was omitted altogether. There was no information about imaging modalities used to assess the extent of disease, the rationale for conversion from mastectomy to breast-conserving therapy, preoperative tumour localization, axillary management, and radiotherapy or BRCA gene status [24].

The NSABP B-27 trial reported that anthracycline-based regimens with the addition of taxane were associated with higher pCR rates and better local control [25]. In our study, a total of 87.6% patients and 89.7% of those treated with BCS had received anthracyclines + taxane. The 8-year isolated breast recurrence rate was 4.8% in the BCS group. The results can be compared to the study by Fowble *et al* [26], who reported the 10-year results of BCS in early-stage cancer, which reported isolated breast recurrence of 6% at 5 years and 16% at 10 years.

In our study, after adjusting for age, cT stage, cN stage, PCR (ypT0/is, N0) and radiotherapy, no statistically significant difference was observed in LRFS, DDFS, RFS and OS between BCS and mastectomy groups. A single large retrospective cohort study from India with 5 years follow-up by Parmar *et al* [27] reported better RFS and lower recurrence in the BCS group, suggesting the feasibility of BCS in LABC in the NACT setting. Several other studies have also reported similar findings, establishing the oncologic safety of BCS in LABC (Table 5).

This study has some potential limitations. First, this was a retrospective study, subject to some inherent biases like different socioeconomic status, comorbidities and loss of follow-up among patients who had a poor outcome. Additionally, all HER2-positive patients did not receive targeted therapy, which restricts the response assessment in this group of patients. The current availability of biosimilar trastuzumab has overcome the affordability issue significantly in developing countries. However, the strength of this study lies in the single institution design, which allowed uniformity in all facets.

## Conclusion

The result of this study demonstrates that, following NACT, rates of LRFS, DDFS, RFS and OS are comparable between BCS and mastectomy. The importance of a multidisciplinary approach with adequate chemotherapy (anthracycline and taxane) and good localisation technique should be considered before offering BCS in LABC patients. These results support BCS as a safe and effective alternative to mastectomy in a selected group of LABC patients.

## Conflicts of interest

The authors declare no conflict of interest for this study.

## Funding disclosure

No external funding received for the study.

## Figures and Tables

**Table 1.** Demographics and histopathological feature (*n* = 411).

Parameters	Results
Age (median, IQR)	49.2 (43, 56)
BMI (median, IQR)	26 (23.1, 29.7)
Type of cancer (*n*, %)	
Invasive ductal cancer	396 (96.3)
Invasive lobular cancer	12 (2.9)
Others	3 (0.8)
Clinical T stage	
T2	24 (5.8)
T3	124 (30.1)
T4	263 (64.1)
Clinical N stage	
N0	37 (9)
N1	269 (65.4)
N2	62 (15.1)
N3	43 (10.5)
Grade	
Grade 1	13 (3.2)
Grade 2	139 (33.8)
Grade 3	259 (63)
Estrogen receptor	
Positive	272 (66.2)
Negative	139 (33.8)
Progesterone receptor	
Positive	189 (56)
Negative	222 (45)
HER 2 receptor	
Positive	148 (36)
Negative	208 (50.6)
Equivocal	55 (13.4)
Tumour subtype (*n* = 356)	
ER/PR positive, HER 2 negative	147 (41.3)
ER/PR positive, HER 2 positive	100 (28.1)
ER/PR negative, HER 2 positive	48 (13.5)
Triple-negative	61 (17.1)
Type of chemotherapy	￼
Anthracycline + taxane	360 (87.6)
Anthracycline only	31 (7.5)
Others	20 (4.9)
Chemotherapy all cycles completed	
Yes	396 (96.35)
No	15 (3.65)
Breast surgery	
Breast conservation + AD	146 (35.5)
MRM	265 (64.6)
Pathological complete response	
Yes	71 (17.3)
No	340 (82.7)
Radiotherapy	
Received	398 (96.8)
Not received	13 (3.2)
Hormonal therapy (*N* = 294)	
Received	285 (96.9)
Not received	9 (3.1)
Trastuzumab (*N* = 148)	
Received	38 (25.7)
Not received	108 (74.3)

**Table 2.** Comparison between two groups (BCS versus mastectomy).

Parameters	BCS	Mastectomy	U/x^2^	*p*
Age (Median, IQR)	45.1 (38.9, 52)	50 (45, 58)	12,958	<0.01
Clinical T stage				
T2	19 (13)	5 (1.9)		
T3	51 (34.9)	73 (27.5)	26.7	<0.01
T4	76 (52.1)	187 (70.6)		
Clinical N stage				
N0	19 (13)	18 (6.8)		
N1	85 (58.2)	184 (69.4)	9.8	0.02
N2	29 (19.9)	33 (12.5)		
N3	13 (8.9)	30 (11.3)		
Grade	￼			
Grade 1	5 (3.4)	8 (3)		
Grade 2	44 (30.1)	95 (35.8)	1.3	0.49
Grade 3	97 (66.5)	162 (61.1)		
Estrogen receptor				
Positive	95 (65.1)	177 (66.8)	0.12	0.74
Negative	51 (34.9)	88 (33.2)		
Progesterone receptor				
Positive	86 (58.9)	136 (51.3)	2.17	0.14
Negative	60 (41.1)	129 (48.7)		
HER 2 receptor				
Positive	43 (29.5)	105 (39.6)		
Negative	79 (54.1)	129 (48.7)	4.8	0.09
Equivocal	24 (16.4)	31 (11.7)		
Tumour subtype (*n* = 356)				
ER/PR positive, HER 2 negative	55 (45.1)	92 (39.3)		
ER/PR positive, HER 2 positive	33 (27)	67 (28.6)	5.26	0.15
ER/PR negative, HER 2 positive	10 (8.2)	38 (16.2)		
Triple negative	24 (19.7)	37 (15.8)		
Type of chemotherapy				
Anthracycline + taxane	131 (89.7)	229 (86.4)		
Anthracycline only	9 (6.2)	22 (8.3)	0.95	0.62
Others	6 (4.1)	14 (5.3)		
Chemotherapy all cycles completed				
Yes	143 (97.9)	253 (95.5)	1.6	0.27
No	3 (2.1)	12 (4.5)		
Pathological complete response				
Yes	33 (22.6)	38 (14.3)	4.49	0.04
No	113 (77.4)	227 (85.7)		
Radiotherapy				
Received	145 (99.3)	253 (95.5)	0.03 (Fisher exact)	
Not received	1 (0.7)	12 (4.5)		
Hormonal therapy (*N* = 294)				
Received	105 (96.3)	175 (94.6)	0.45	0.5
Not received	4 (3.7)	10 (5.4)		
Trastuzumab (*N* = 148)				
Received	10 (23.3)	28 (26.7)	0.18	0.68
Not received	33 (76.7)	77 (73.3)		

**Table 3.** Type of recurrence.

Recurrences	BCS (*n* = 146)	Mastectomy (*n* = 265)	Total (*n* = 411)
Local	13 (8.9%)	22 (8.3%)	35 (8.5%)
Breast/chest wall	7 (4.8%)	5 (1.9%)	12
Locoregional	6 (4.1%)	17 (6.4%)	23
Distant	36 (24.7%)	106 (40%)	142 (34.5%)

**Table 4.** Cox regression survival analysis (BCS versus mastectomy).

	Crude HR (95% CI)	^a^Adjusted HR (95%CI)
LRFS	1.02 (0.51, 2.01)	1.1 (0.53, 2.3)
RFS	0.70 (0.50, 1)	0.80 (0.55, 1.17)
DDFS	0.57 (0.39, 0.84)	0.67 (0.45, 1.01)
OS	0.58 (0.36, 0.93)	0.69 (0.41, 1.14)

a Adjusted for age, cT stage, cN stage, PCR, RT

**Table 5.** Studies on the oncologic safety of BCS in LABC.

Study	Year	Sample size	Population	Study design	Median FU (months)	5 years RFS (BCS versus MRM)	5 years OS (BCS versus MRM)
Present study	2023	411	LABC	Retrospective	61	63.9% versus 57.9% (*p* = 0.05)	79.3% versus 71.5% (*p* = 0.03, NS in Cox regression)
Barranger et al [28]	2015	119	LABC	Retrospective	41.1	74% versus 49% (p = NS)	77% in both
Levy *et al* [22]	2014	284	LABC	Retrospective	75	-	90% versus 76% (*p* = 0.13)
Cho *et al* [29]	2013	593	Stage 2 and 3 breast cancer	Retrospective	45.9	85.9% versus 74.6% (*p* = 0.90)	89.1% versus 84.2% (*p* = 0.217)
Sweeting *et al* [21]	2011	122	Stage 2 and 3 breast cancer (young women <45 years)	Prospective database	76	82% versus 58% (*p* = 0.03)	88 versus 61% (*p* = 0.004)
Parmar *et al* [27]	2006	664	LABC	Retrospective	30	62% versus 37% (*p* < 0.01)	-
